# Natural Language Processing Algorithm to Extract Multiple Myeloma Stage From Oncology Notes in the Veterans Affairs Healthcare System

**DOI:** 10.1200/CCI.23.00197

**Published:** 2024-07-22

**Authors:** Sergey D. Goryachev, Cenk Yildirim, Clark DuMontier, Jennifer La, Mayuri Dharne, J. Michael Gaziano, Mary T. Brophy, Nikhil C. Munshi, Jane A. Driver, Nhan V. Do, Nathanael R. Fillmore

**Affiliations:** ^1^ Massachusetts Veterans Epidemiology Research and Information Center (MAVERIC), Boston, MA; ^2^ VA Boston Healthcare System, Boston, MA; ^3^ VA Boston Cooperative Studies Program, Boston, MA; ^4^ New England Geriatrics Research, Education and Clinical Center, VA Boston Healthcare System, Boston, MA; ^5^ Division of Aging, Brigham and Women's Hospital, Boston, MA; ^6^ Divison of Population Sciences, Dana-Farber Cancer Institute, Boston, MA; ^7^ Harvard Medical School, Boston, MA; ^8^ Boston University School of Medicine, Boston, MA; ^9^ Department of Medical Oncology, Dana-Farber Cancer Institute, Boston, MA

## Abstract

**PURPOSE:**

Stage in multiple myeloma (MM) is an essential measure of disease risk, but its measurement in large databases is often lacking. We aimed to develop and validate a natural language processing (NLP) algorithm to extract oncologists' documentation of stage in the national Veterans Affairs (VA) Healthcare System.

**METHODS:**

Using nationwide electronic health record (EHR) and cancer registry data from the VA Corporate Data Warehouse, we developed and validated a rule-based NLP algorithm to extract oncologist-determined MM stage. To that end, a clinician annotated MM stage within over 5,000 short snippets of clinical notes, and annotated MM stage at MM treatment initiation for 200 patients. These were allocated into snippet- and patient-level development and validation sets. We developed MM stage extraction and roll-up algorithms within the development sets. After the algorithms were finalized, we validated them using standard measures in held-out validation sets.

**RESULTS:**

We developed algorithms for three different MM staging systems that have been in widespread use (Revised International Staging System [R-ISS], International Staging System [ISS], and Durie-Salmon [DS]) and for stage reported without a clearly defined system. Precision and recall were uniformly high for MM stage at the snippet level, ranging from 0.92 to 0.99 for the different MM staging systems. Performance in identifying for MM stage at treatment initiation at the patient level was also excellent, with precision of 0.92, 0.96, 0.90, and 0.86 and recall of 0.99, 0.98, 0.94, and 0.92 for R-ISS, ISS, DS, and unclear stage, respectively.

**CONCLUSION:**

Our MM stage extraction algorithm uses rule-based NLP and data aggregation to accurately measure MM stage documented in oncology notes and pathology reports in VA's national EHR system. It may be adapted to other systems where MM stage is recorded in clinical notes.

## Teaser

Stage is a key factor in assessment of multiple myeloma (MM) risk, but large databases lack the necessary laboratory and pathology data for staging. We developed a rules-based NLP algorithm to extract oncologist-determined MM stage from VA EHR data. The algorithm demonstrated accurate assignment of MM stage when validated against clinician annotation.

## INTRODUCTION

Stage in multiple myeloma (MM) is an essential measure of disease risk, but its measurement in large databases is often lacking.^1,2^ Part of the difficulty in measuring MM stage is its derivation from data spanning serum, blood, and pathologic specimens, in contrast to the tumor size and spread criteria used for solid tumors.^3^ Large registry databases such as the SEER Program and administrative databases such as Centers for Medicare and Medicaid Services lack these data necessary to compute stage. The nationally integrated Veterans Affairs (VA) Healthcare System has access to structured laboratory and pathologic data, but there is still a substantial amount of missingness in the more specialized components of stage, such as beta-2 microglobulin and tumor cytogenetic mutations that comprise the International Staging System (ISS) and Revised International Staging System (R-ISS). For many Veterans using VA, these stage components may have been first measured externally in non-VA health systems, and therefore not recorded in VA's central database. Failure to include MM stage in any analysis could introduce bias and confounding because of imbalances in disease risk across exposure groups.

**CONTEXT**

**Key Objective**
Since multiple myeloma (MM) stage is often lacking in the structured data, could natural language processing (NLP) extract it from oncology notes?
**Knowledge Generated**
We developed a rule-based NLP method to extract oncologists' documentation of stage in the clinical notes of patients with MM who were treated in the Veterans Affairs Healthcare System. Performance of the algorithm was high when validated against clinician-annotated stage.
**Relevance *(F.P.-Y. Lin)***
The extraction of MM stages from oncology notes using a rule-based NLP algorithm provides an automatable and scalable solution, ensuring consistent information provision on myeloma stages for research and clinical analytics.*
*Relevance section written by *JCO Clinical Cancer Informatics* Associate Editor Frank P.-Y. Lin, PhD, MBChB, FRACP, FAIDH.

Oncologists often synthesize and interpret the disparate data sources to document stage in clinical notes. Fortunately, VA data have the unique advantage of having access to such clinical notes from its national electronic health record (EHR). However, manual review of these notes would be impractical and susceptible to user error in retrospective studies involving larger cohorts from the full population of nearly 20,000 VA patients with MM.^4,5,^

In the current study, we aimed to develop and validate a natural language processing (NLP) method to extract oncologists' documentation of stage in the clinical notes of patients with MM who were treated in VA. We developed an NLP method to extract oncologist-determined stage from short snippets of notes as well as an algorithm to assign MM stage at MM treatment initiation at the patient level, and we validated these algorithms on held-out validation sets.

## METHODS

### Data Sources and Study Population

This study is based on nationwide EHR and cancer registry data from the VA Corporate Data Warehouse (CDW). The CDW collects clinical, billing, and EHR information from Veterans treated in VA facilities throughout the United States.^6^ It also includes national cancer registry data from the VA Cancer Registry System (VACRS), which includes information on cancer diagnosis and treatment compiled by the local cancer registry staff at each of the 132 VA Medical Centers that diagnose and/or treat Veterans with cancer.^7,8^ Note that MM stage is not captured in the VACRS, and therefore the VACRS was used only for cohort identification, not MM stage extraction.

The study population consists of patients with MM treated at the VA between 1999 and 2019. Patients were identified using methods that integrate diagnosis and treatment information from the EHR and VACRS as described and validated in our previous work.^9^ This study was approved by the VA Boston Healthcare System Institutional Review Board.

### Creation of Annotated Data Sets

We created two annotated data sets: a snippet-level data set used for development and validation of the NLP method to extract oncologist-determined MM stage, and a patient-level data set used for overall ascertainment of a patient's MM stage as determined by their oncologist at a particular timepoint.

The snippet-level data set was created as follows. Within the study cohort of patients with MM, we extracted all clinical notes that matched a broad pattern for potential references to MM stage, and we created short snippets of 100 characters before and 200 characters after each pattern match within these clinical notes. The regular expression for this pattern can be found in the Data Supplement. We randomly sampled 5,207 snippets, and we prepared an annotation interface to annotate these snippets using Label Studio.^10^ Annotation was conducted by a board-certified clinician (C.D.) using the annotation guidelines described below. After annotation, annotated snippets were randomly allocated into a snippet-level development set (80%, n = 4,166) and a snippet-level validation set (20%, n = 1,041).

The patient-level data set was created as follows. We randomly sampled 200 patients with MM. For each patient, all clinical notes between 1999 and 2019 were displayed in a searchable interface within Label Studio. A board-certified clinician (C.D.) annotated MM stage at MM treatment initiation using the annotation guidelines below. Date of MM treatment initiation was determined by occurrence of the first MM-specific treatment code (eg, bortezomib or lenalidomide) after diagnosis, using our validated algorithm selecting for patients initiating MM within VA (93% positive predictive value).^9^ Date of treatment initiation was chosen over date of diagnosis to represent the most relevant scenario in which staging information would be informative, as well as better ensure selection of patients being treated and followed within VA. Annotated patients were randomly allocated into a patient-level development set (50%, n = 100) and a patient-level validation set (50%, n = 100).

**FIG 1.**
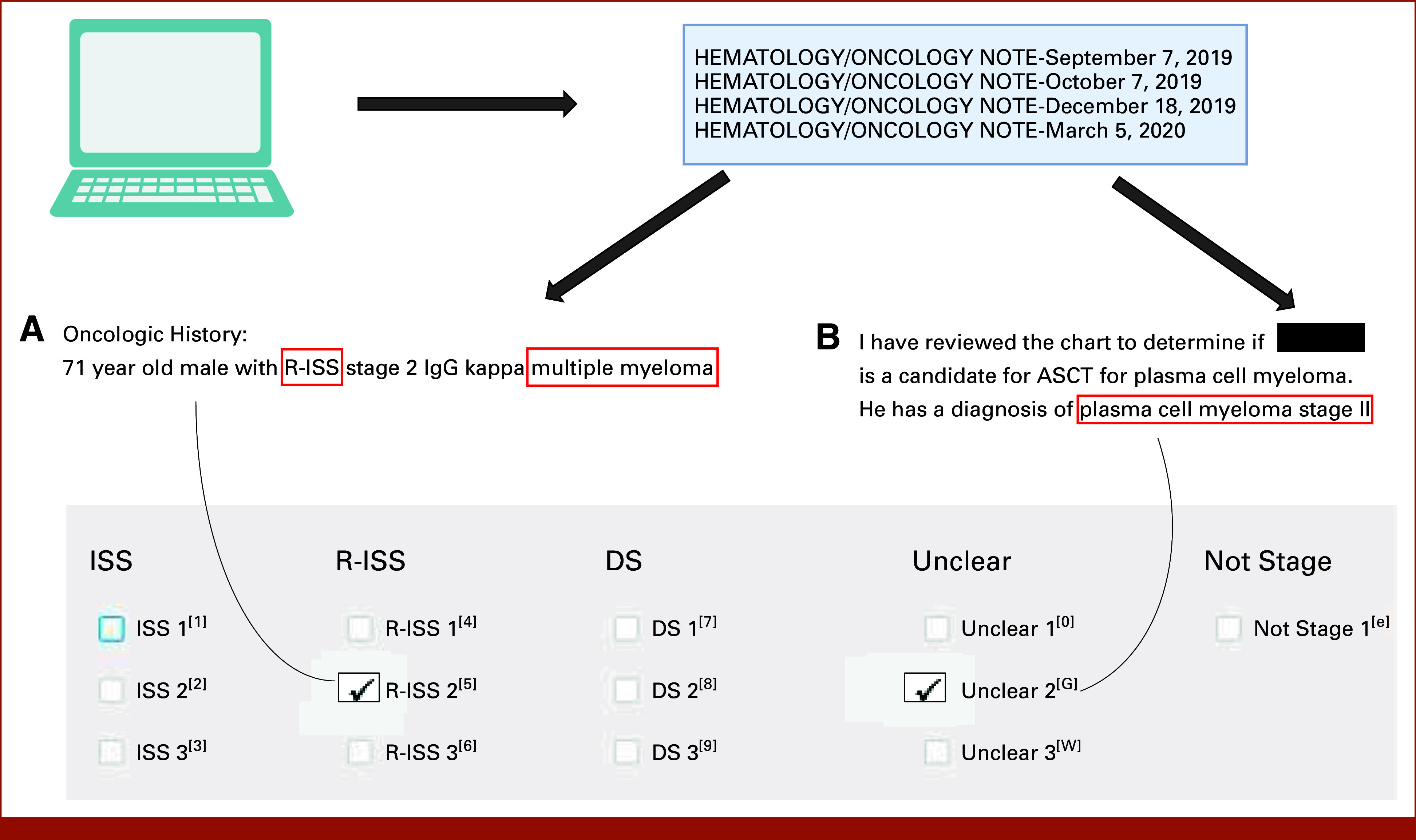
Annotation process: oncology and related notes were extracted from the VA Corporate Data Warehouse. Snippets of notes potentially containing stage information were presented to a clinician for annotation. (A) An annotation for clear stage. (B) Unclear stage. ASCT, autologous stem cell transplant; DS, Durie-Salmon; IgG, immunoglobulin G; ISS, International Staging System; R-ISS, Revised International Staging System; VA, Veterans Affairs.

Annotation guidelines were as follows. For snippet-level annotation, the clinician annotated the following for each snippet: (1) whether the words in the snippet pertained to the patient's MM stage (yes/no); (2) if yes, whether there was a clear staging system documented (Durie-Salmon [DS], ISS, R-ISS); and (3) the specific stage (I/II/III) documented (Table 1; Fig 1). If multiple stages were present, then the clinician marked all of these. However, if both a stage(s) documented as clearly belonging to one of the three MM staging systems and a stage(s) documented without a clear staging system were present, then the clinician only annotated the clearly documented staging system(s). For patient-level annotation, this procedure was the same, except that the annotations pertained to each patient's stage at MM treatment initiation on the basis of the global picture provided by all clinical notes. For both snippet-level and patient-level annotations, the clinician did not attempt to interpret or incorporate text referring to laboratory variables including in the staging systems (eg, albumin or beta-2 microglobulin); only language pertaining to the staging systems themselves were used for annotation.

**TABLE 1.** Staging Systems Annotated by the Clinician in Each Clinical Note Snippet

R-ISS	ISS	DS^a^	Unclear Stage^b^	Not Stage
I	I	I	I	Marked if no MM stage found
II	II	II	II	
III	III	III	III	

Abbreviations: DS, Durie-Salmon; ISS, International Staging System; MM, multiple myeloma; R-ISS, Revised International Staging System.

a For simplicity, we did not distinguish between DS subclasses A and B.

b Stage value was annotated under unclear if a stage was documented in the clinical note but the name of the system used was not documented.

### Development and Validation of Snippet-Level Stage Extraction

We developed a rule-based NLP algorithm to extract MM stage documented by oncologists using the snippet-level development set. We made use of the Apache Unstructured Information Management Architecture NLP framework^11^ to compose a pipeline of regular expression-based rules to identify MM mentions, staging systems, stage values, irrelevant diseases, and general tokens in the note text. We iteratively improved the algorithm on the basis of evaluation measures in the snippet-level development set until evaluation measures stabilized at an acceptable level.

After the development of the final snippet-level NLP algorithm was complete, we evaluated the performance of the NLP algorithm at the snippet level using the validation set of annotated snippets. The evaluation was done separately for different staging systems: R-ISS, ISS, DS, unclear staging system, and not stage. We evaluated standard measures (precision, recall, and F1 score) on the basis of contingency tables.

### Development and Validation of Patient-Level Stage Assignment

Each patient typically has multiple notes with multiple snippets that contain text referring to MM stage. However, for downstream applications, a single stage value for each patient is typically desired, most commonly at the time of diagnosis or treatment initiation. Therefore, we developed and validated simple algorithms to roll-up snippet-level stage labels to the patient level at the time of treatment initiation. Using data from the patient-level development set, we developed simple algorithms to roll-up snippet-level stage assignments to the patient level. We iteratively modified these algorithms on the basis of standard evaluation measures (precision, recall, and F1 score) in the development set. After the roll-up algorithms were finalized, we evaluated the same evaluation measures in the patient-level validation set.

## RESULTS

### Final Snippet-Level Algorithm and Its Validation

The snippet-level MM stage extraction algorithm was developed on the basis of 5,207 snippets in the snippet-level development set. The final rule-based NLP algorithm consists of (1) span labeling rules, (2) value normalization rules, and (3) association rules to combine the foregoing to obtain the final MM stage assignments for each snippet. Span labeling rules include rules to label stage values (eg, 3 or III) and staging system categories (eg, ISS). In addition, there are rules to identify diseases other than MM, such as chronic kidney disease, for which stage may also be reported. Value normalization rules map different stage value spellings such as roman numerals into standardized Arabic numeric stage values (eg, III is mapped to 3), and staging system spelling variants into standardized staging system names (eg, International Staging System is mapped to ISS). Finally, association rules are as follows. Spans that overlap a span previously labeled with the same label are discarded, with priority determined by the order of rule evaluation. Stage value spans in proximity to spans mentioning diseases other than MM are discarded to reduce the number of false-positive stage values associated with non-MM diseases. Among the remaining labeled spans, stage value spans are associated with staging system spans–based additional patterns related to proximity and linguistic content. If no staging system is associated with a stage value, the staging system is set to unclear stage. After all rules are applied, the resulting stage value-staging system pairs are assigned to the snippet as its stage labels. It is possible that more than one stage label could be present, for example, it is possible for a single snippet to have both R-ISS 2 and ISS 3 present. The detailed span labeling rules, value normalization rules, and association rules are available in the Data Supplement.

After the final snippet-level MM stage extraction algorithm was complete, we evaluated its performance relative to 1,041 snippets in the snippet-level validation set, using standard measures. Precision, recall, and F1 were uniformly high in the held-out snippet-level validation set, ranging between 0.92 and 0.99 (Table 2). Specifically, precision (ie, positive predictive value) was 0.99, 0.97, 0.93, and 0.98 for R-ISS, ISS, DS, and unclear stage, respectively. Recall was 0.99, 0.98, 0.94, and 0.92, respectively, and the F1 score was 0.99, 0.98, 0.93, and 0.95, respectively. Results were similarly strong across strata defined by the geographic region of the hospital where the snippet's note was written (Data Supplement, Table S1).

**TABLE 2.** Performance of NLP Algorithm to Extract MM Stage at the Snippet Level on the Basis of the Snippet-Level Validation Set (1,041 snippets)

Staging System	TP	TN	FP	FN	Precision	Recall	F1 Score
R-ISS	204	832	2	3	0.99	0.99	0.99
ISS	380	642	10	9	0.97	0.98	0.98
Durie-Salmon	165	852	13	11	0.93	0.94	0.93
Unclear stage	313	693	6	29	0.98	0.92	0.95

Abbreviations: FN, false negative; FP, false positive; ISS, International Staging System; MM, multiple myeloma; NLP, natural language processing; R-ISS, Revised International Staging System; TN, true negative; TP, true positive.

### Final Patient-Level Algorithm and Its Validation

The algorithm to assign MM stage at the patient level was developed on the basis of 100 patients in the patient-level development set. From these development-set patients, we identified all available clinical notes (169,315 notes). We applied the final snippet-level algorithm to extract MM stage from all snippets created from these notes, resulting in 4,542 snippet-level labels. By comparing these extracted snippet-level labels to the clinician's annotations of stage at treatment initiation, we developed the following final patient-level algorithm to assign MM stage at treatment initiation (Table 3).

**TABLE 3.** Performance of the Final Algorithm to Extract MM Stage at the Patient Level at MM Treatment Initiation on the Basis of the Patient-Level Validation Set (100 patients)

Staging System	TP	TN	FP	FN	Precision	Recall	F1 Score
R-ISS	11	88	1	0	0.92	1.00	0.96
ISS	25	73	1	1	0.96	0.96	0.96
Durie-Salmon	19	78	2	1	0.90	0.95	0.92
Unclear stage	18	78	3	1	0.86	0.95	0.90

Abbreviations: FN, false negative; FP, false positive; ISS, International Staging System; MM, multiple myeloma; R-ISS, Revised International Staging System; TN, true negative; TP, true positive.

First, we include only hematology/oncology notes and pathology reports within 1 year before and after the first MM treatment date for each patient, to increase specificity for notes pertaining to MM stage at MM treatment initiation. We evaluated several assessment windows and found that 1 year before/after had the best performance in the development set. We applied the snippet-level NLP to these notes. Second, for each staging system (R-ISS, ISS, DS, and unclear stage), we locate the clinical note closest to the date of MM treatment initiation that contains a snippet with a stage value for this staging system. We assign that stage value to the patient for this staging system. If no included snippets contain a value for the staging system, no value is assigned; if there are no values for any of the staging systems, then unclear stage is assigned. If no included snippets contain any stage information, no stage is assigned to the patient (ie, stage would be missing for that patient).

After the final patient-level MM stage assignment algorithm was complete, we evaluated its performance relative to 100 patients in the patient-level validation set (Data Supplement, Table S2). Precision was 0.92, 0.96, 0.90, and 0.86 for R-ISS, ISS, DS, and unclear stage, respectively. Recall was 1.00, 0.96, 0.95, and 0.95, respectively. The F1 score was 0.96, 0.96, 0.92, and 0.90, respectively. Comparison with laboratory test–derived ISS stage revealed that in 15% of patients, no laboratory test–derived ISS stage was available but NLP-derived stage was available, and in an additional 10% of patients, R-ISS stage was available from NLP but only ISS stage was available from laboratory test data (Data Supplement, Table S3).

## DISCUSSION

After development and testing against thousands of clinician annotations, our MM stage extraction algorithm combines NLP and data aggregation to accurately measure oncologist-determined MM stage from clinical notes documented in VA's national EHR. Our MM stage extraction algorithm makes the most of the unique data available within the nationally integrated VA Healthcare System to measure a clinically important variable that is lacking in structured data.

Other examples have leveraged NLP to extract from VA's robust EHR data measures related to homelessness in US Veterans,^12^ phenotypes of patients with inflammatory bowel disease,^13^ and clinical characteristics associated with congestive heart failure.^14^ In oncology, other groups using other data sets have applied NLP-based methods that leverage existing ontologies or terminology on which to map text fragments from unstructured data related to treatment regimens, cancer toxicities, and even MM-related measures.^15-17^ The advantages of using existing, broad ontology/terminology frameworks in NLP extraction and mapping needs to be weighed against their potential lack of adaptability to specific data sets, lack of specificity for certain phenotypes, and heterogeneous, often insufficient validation of individual variables.^18^ For example, Loda et al^17^ evaluated in their local University Hospital in Germany the performance of NLP mapping to an ontology framework in only 10 cases of ISS stage III MM. Rather than seeking to apply a broad framework to develop NLP extraction of multiple MM measures, we focused our development of NLP specifically to extract MM stage from VA unstructured data—similar to the approach by Ryu and Zimolzak^19^ to NLP extraction of monoclonal gammopathy in short text reports. We believe this focused approach guiding the development and iteration of rules specific to VA unstructured data allowed us to achieve high performance in the hold-out validation set across all MM staging systems. More direct comparisons of the performance between focused NLP tailored to a specific variable/phenotype and broad NLP using ontology/terminology frameworks are needed. A potential criticism against NLP-extracted stage is that it is less objective than assigning stage directly from laboratory and pathology data. However, there is an advantage of measuring the treating oncologists' documented stage over an investigator's retrospective assignment, given that the oncologist had the best expertise and patient-level knowledge.^20^ Moreover, measuring oncologists' documented stage not only indirectly captures missing variables needed to compute MM stage that are not readily available in structured data (such as cytogenetics/fluorescent in situ hybridization results), but also the clinical expertise to accurately interpret these variables and assign the appropriate value.^21^ For example, an experienced oncologist may be able to better interpret or even impute a high beta-2 microglobulin value for staging purposes in a patient with end-stage renal disease related to myeloma.^22^ An oncologist could best assess the presence and severity of bone disease from available imaging data (eg, a negative magnetic resonance imaging in absence of skeletal surveys and computed tomography data). In practical application, our NLP-extracted stage algorithm can be combined with structured stage extraction to leverage all aspects of VA data and minimize missingness of stage in research. We have recently demonstrated such a combined approach in a recently published study investigating intensity of MM therapy by frailty status, in which MM stage was measured and assigned to each patient through a hierarchical approach that preferred R-ISS from NLP if available, followed by ISS from either NLP or from direct calculation using beta-2 microglobulin and albumin values available in structured laboratory data.^23^

Our patient-level MM stage roll-up algorithm is designed to extract stage at initial treatment, given the importance of initial staging in risk stratification, prognosis, and treatment planning.^23^ However, our algorithm can be readily adapted to extract stage at other time points (eg, restaging at first relapse or progression), depending on the needs of the research team, by recentering the clinical note selection window. Alternative approaches to this problem are possible. For example, machine learning–based NLP could be used instead of the rules-based approach that we used, and in certain, cases machine learning might yield superior performance when extracting MM-specific or other cancer-based measures.^24^ We opted for a rule-based approach, given the circumscribed, well-defined MM staging terminology that facilitated defining simple rules related to the wording, grammar, and syntax found in oncologists' notes when they document stage. Moreover, with a rule-based approach, errors detected during development were readily fixable, given that the clinician and analysts could modify rules on the basis of their subject matter expertise.

For example, one of the keyword rules used to identify Durie-Salmon or DS was falsely capturing DS from double-strength antibiotics and denoting this descriptor as MM stage. This error was easily corrected by modifying the rules to avoid labeling DS as stage when it appears near words such as “Bactrim” or other antibiotic names. By contrast, errors made by machine learning–based NLP algorithms are more difficult to debug, given that they train against the data without understanding terminology, grammar, or syntax. In the above DS antibiotic example, a machine learning–based NLP algorithm may falsely identify DS as stage; in that case, locating which part of the algorithm to fix would be difficult, and the typical solution would be to provide more clinician-annotated data against which to further train the algorithm, which is typically more time-consuming than adding a new rule. The choice of whether to use rule-based, machine learning, or a mix of both NLP approaches depends on the variable(s) needed to be extracted and the data sources that contain them.^24^

There are limitations to our study. Our algorithm aggregates notes from 1 year before and after initial MM treatment and can identify multiple stages within this time frame; although we use rules to increase the likelihood that the initial stage is assigned to the patient, there is no guarantee that stage at a later time point or an incorrect stage altogether (eg, documented by the oncologist on the basis of incomplete data) is assigned. However, our algorithm performed with high precision against clinician-annotated initial stage, and it is very rare—reinforced by our review of cases—for oncologists to restage or document different stages in the year after initial treatment. In addition, our algorithm does not include the latest revision of the R-ISS (R2-ISS),^25^ since this was only recently released and has not yet been widely adopted within the VA system. Our algorithm can be readily adapted to extract this and other iterations of staging systems once data on their application in clinical practice are more widely available. Our algorithm includes outdated systems no longer in widespread use such as DS because it is intended to be applied over a broad time span of data availability, including in earlier eras when DS was more widely used. In downstream applications, the algorithm can be modified to preferentially assign stage from the most up-to-date staging system available for each patient (eg, prefer R-ISS over DS if both are documented and conflicting).

In conclusion, our algorithm to extract MM stage can accurately measure this variable on the basis of data in clinical notes, reducing the missingness of this important measure of MM risk. Researchers at the VA can use our MM stage extraction algorithm to incorporate MM stage as a predictor or covariate in future analyses seeking to evaluate outcomes in Veterans treated with MM therapies, and researchers outside the VA can adapt our algorithm to their respective systems. Our example showcases the new horizon of leveraging all available data to phenotype patients with cancer, which will carry further applications in the coming years as multiple layers of genetics and omics data become more widely available to investigators in the form of structured and unstructured data.^26,27^

## Data Availability

The data underlying this article were accessed from the VA Corporate Data Warehouse. The derived data generated in this research may be shared on reasonable request to the corresponding author as permitted by VA policy.
